# Dyslipidemia in Patients with a Cardiovascular Risk and Disease at the University Teaching Hospital of Yaoundé, Cameroon

**DOI:** 10.1155/2017/6061306

**Published:** 2017-01-09

**Authors:** Vicky Jocelyne Ama Moor, Sylvie Ndongo Amougou, Sebastien Ombotto, Felicien Ntone, Doriane Edna Wouamba, Bernadette Ngo Nonga

**Affiliations:** ^1^Department of Biochemistry, Faculty of Medicine and Biomedical Sciences, University of Yaoundé I, Yaoundé, Cameroon; ^2^Department of Internal Medicine, Faculty of Medicine and Biomedical Sciences, University of Yaoundé I, Yaoundé, Cameroon; ^3^Institute of Medical Technology, Yaoundé, Cameroon; ^4^Department of Surgery, Faculty of Medicine and Biomedical Sciences, University of Yaoundé I, Yaoundé, Cameroon

## Abstract

*Objective*. To determine the frequency of lipid abnormalities in patients with a cardiovascular risk and disease at the University Teaching Hospital (UTH) of Yaoundé.* Materials and Methods*. We conducted a cross-sectional study from 1 March to 31 May 2015 at the UTH of Yaoundé. We included all patients seen in the outpatient department with a diagnosis of a cardiovascular disease or a risk factor for cardiovascular disease. Patients who accepted to participate in the study were asked to answer a questionnaire; after that a blood sample was taken for lipid profile. An informed consent was signed by all the participants and the study has received approval from the national ethic committee.* Results*. We recruited 264 patients of which 119 were men and 145 were women with a sex ratio of 0.82. Mean age was 61.36 years. The frequency of lipid profiles abnormalities was as follows: low HDL cholesterol (44.3%), hypertriglyceridemia (18.9%), high LDL cholesterol (3.8%), and high total cholesterol 3.4%). Hypertriglyceridemia was strongly associated with type 2 diabetes mellitus.* Conclusion*. Low levels of HDL cholesterol and hypertriglyceridemia are more prevalent in our study population. More studies are needed to confirm this finding in our environment.

## 1. Introduction

Cardiovascular diseases involve the cardiovascular system: heart, blood vessels, and the circulatory system [1]. They represent the first cause of invalidity in developed countries. According to World Health Organization, the prevalence of cardiovascular diseases will double by 2020 and will rank before the HIV/AIDS infection [2].

One of the major risk factors of cardiovascular diseases is arteriosclerosis which is secondary to the excess of LDL cholesterol. Clinical manifestations of arteriosclerosis are found in coronary artery disease, ischemic stroke, and peripheral vascular occlusive diseases. Dyslipidemia is a metabolic abnormality leading to a persistent increase in the plasmatic concentration of cholesterol and triglycerides. There are currently three types of dyslipidemia, hypercholesterolemia, hypertriglyceridemia, and mixed hyperlipidemia, due to elevation of both cholesterol and triglycerides [3]. Dyslipidemia is a common cause of morbidity all over the world, and the most common form is hypercholesterolemia defined as a total cholesterol level above 5,0 mmol/L or 190 mg/L. One-third of ischemic heart diseases in the world are secondary to hypercholesterolemia, and it is estimated that hypercholesterolemia is responsible for 2.6 million (4,5%) deaths in the world [4]. In 2008, the worldwide prevalence of hypercholesterolemia in western countries was 39% in males and 40% in females [5]. In Cameroon, very few studies have been conducted to determine the frequency of the dyslipidemias in our environment.

The objective of this study was to determine the prevalence of lipid profile abnormalities in patients presenting with a cardiovascular disease or risk factor at the University Teaching Hospital of Yaoundé, Cameroon.

## 2. Patients and Methods

### 2.1. Patients

We carried out a cross-sectional study from March to May 2015 at the University Teaching Hospital (UTH) of Yaoundé. The study included all patients with a cardiovascular disease seen at the UTH during that period: ischemic heart disease, stroke, peripheral vascular disease, hypertension, and cardiomyopathies; we also included patients with a risk factor for cardiovascular disease such as diabetes mellitus. All patients were informed about the protocol and if they accepted to participate in the study, they were asked to sign a consent before enrollment. We excluded patients with an incomplete file and hemolyzed blood serum and those who refused to participate in the study.

### 2.2. Methods

Patients were given a questionnaire administered by the investigators. The questionnaire was first administered to 16 nonmedical persons accompanying patients at the emergency department as a pretest and validated. Collected data were sociodemographic information (age and sex,) and cardiovascular risk factors and diseases. Blood was drawn by the same person who respected the appropriate recommendations before the determination of plasma lipid's abnormalities.

The first phase was the preanalytical phase: patients were asked to fast for 12 hours before blood was drawn. The elbow was cleaned and blood was taken in a tube without anticoagulant; we use a loose vein occlusion to minimize the risk of hemolysis. Specimens were taken to the laboratory in less than 20 minutes after the procedure in a specific box.

The tubes were centrifuged at 3000 rpm for 5 minutes before preservation.

Our judgment criteria for a dyslipidemia were as follows [6]:

Total cholesterol > 2,70 g/L
HDL cholesterol < 0,44 g/L
LDL cholesterol > 1,88 g/L
Triglycerides > 1,50 g/L

Analysis was done using the CYAN START photometer of the CYPRESS Laboratory (Langdorp, Belgium). Total cholesterol, HDL cholesterol, and triglycerides were determined using an enzymatic method found in commercial kits of the CYPRESS Laboratory (Langdorp, Belgium); LDL cholesterol was calculated using the formula of Friedewald (1972) [7]. Results were validated using a quantitative control serum.

### 2.3. Statistical Analysis

Data were encoded using the CS Pro software version 4.1. Statistical analysis was done using Epi Info 3.54 software and represented in tables using the Microsoft Excel 2010 software. Statistical test used was chi square. Difference was found to be significant if *P* < 0.05.

### 2.4. Ethical Considerations

The study has obtained ethical clearance from the national ethic committee of Cameroon (number CEI-UD/246/02/2015/T). Informed consent was obtained before enrollment in the study.

## 3. Results

### 3.1. Sociodemographic Characteristics of the Patients

During that period, 596 patients with a cardiovascular disease or risk factors were seen at the UTH, of which 350 met the inclusion criteria and accepted to participate in the study. Because of poverty, only 264 were able to have a lipid profile done and 86 patients were then excluded because of incomplete file. The population of the study composed of 264 patients of which 119 (45.1%) were men and 145 (54.9%) were women, giving a sex ratio of 0.81. The mean age was 61.36 years with a minimum of 32 years and a maximum of 88 years. The most common age group was between 50 and 60 years and represented 36% of patients.

### 3.2. Risk Factors for Cardiovascular Disease and Life Style

Sedentariness was found in 188 (71%) patients, 142 (53.7%) patients drank alcohol regularly, obesity was found in 74 (27.9%) patients, 19 (7.1%) patients were smokers, hypertension was found in 169 (64%) patients, and 43 (16.3%) patients had diabetes mellitus. The association of hypertension and diabetes mellitus was found in 29 patients (10.98%).

### 3.3. Cardiovascular Pathologies

The most common cardiovascular disease was hypertension found in 169 patients (64%), followed by stroke found in 35 patients (13,25%), ischemic heart diseases in 14 patients (5.3%), and other cardiomyopathies in 16 patients (6.06%). One patient had deep venous thrombosis.

### 3.4. Abnormalities of the Lipid Profile

The results of the lipid profile in the 264 patients are shown in Table 1: only 9 patients (3.4%) had high total cholesterol levels, 10 (3.8%) patients had a higher than normal LDL cholesterol, and 50 (18.9%) had above normal triglycerides levels, giving prevalence of dyslipidemia of 26%.

Mean total cholesterol levels and LDL cholesterol levels were higher in women than in men and this difference was significant (*P* = 0.01 and *P* = 0.03, resp.) (Table 1), while mean HDL cholesterol and triglycerides were also higher in women than in men, but this difference was not statistically significant (Table 2). Based on the definition of dyslipidemia, the most common abnormality was hypertriglyceridemia (18.90%) followed by LDL hypercholesterolemia (3.8%) and total hypercholesterolemia (3.4%). Low level of HDL cholesterol was found in 117 (44.3%) patients (Table 3): 35 patients were on statin medications (anticholesterol drugs); the remaining 82 patients were not on any anticholesterol drugs. Low HDL and low LDL cholesterol were simultaneously present in 37 (31.62%) patients from 117 who had low HDL cholesterol (Table 3). These patients were considered to have low risk lipid profile. If we consider low HDL cholesterol as a dyslipidemia, 149 of the patients were having a lipid abnormality, giving corrected prevalence of 56.4% for a dyslipidemia in our study. Only one patient had high LDL cholesterol and low HDL and he was considered to have high risk lipid profile. In this group, 4 patients (3.4%) had atherogenic dyslipidemia (low HDL and elevated LDL or triglycerides) (Table 3).

From 131 (49.6%) patients who had a normal HDL cholesterol, 32 (24.4%) had also low LDL and triglycerides level, 27 (20.61%) had normal LDL and TG levels, 6 (4.58%) had a normal HDL and an elevated LDL, and 15 (11.45%) had elevated triglycerides. There was no mixed dyslipidemia in this study (Table 4).

Sixteen (6%) patients had a high HDL cholesterol, of which 6 (37.5%) had an elevated LDL and a low triglyceride level, 2 (12.5%) had normal LDL and triglycerides, and one had elevated triglycerides with a low LDL and high HDL (Table 5).

Regarding atherogenic index (total cholesterol/HDL cholesterol), 95 (36%) of our patients had an index of more than 4.5 and 169 (64%) had an index below 4.5.

There was a strong association between diabetes mellitus and hypertriglyceridemia (odds ratio = 2.73 and *P* = 0.003).

Low HDL cholesterol level and hypertriglyceridemia were more frequent in patients with hypertension (74.4% and 84%, resp.). It was the same in patients with diabetes mellitus (11.1% and 18%).

## 4. Discussion

Considering low HDL cholesterol or hypocholesterolemia HDL as a risk factor we have found prevalence of dyslipidemia in our patients with a cardiovascular risk or disease of 56.4%, with 36% of the patients having an elevated atherogenic index of more than 4.5. Dyslipidemia would then be the fourth risk factor for cardiovascular disease as reported by Karaye et al. in Nigeria [8].

Levels of total, HDL, and LDL cholesterol and TG were more elevated in women than in men, although our values were lower than the ones reported by Youmbissi et al. in 2001 [9]. This difference may be due to the difference in the number of patients (we have a larger number of patients) and also Youmbissi et al. studied only patients with hypertension. Thirty-five of the patients were on anticholesterol medications which may have contributed to the increase of the number of patients with low HDL cholesterol and low LDL cholesterol at the same time. High levels of abnormal lipid profile in women compared to men have also been reported in Nigeria [10]. Mean triglycerides levels in our study were lower than the ones reported from Gabon [11] and mean values of HDL cholesterol were lower than the ones reported by Youmbissi et al. from Cameroon [9].

The most common lipid abnormality was low HDL cholesterol, followed by elevated triglycerides and elevated LDL cholesterol, with high level of total cholesterol being the last one. All these values were more elevated in women than in men. This result is different from the one reported by Apetse et al. from Togo who found total hypercholesterolemia followed by LDL hypercholesterolemia and the hypertriglyceridemia to be the most common lipid abnormalities in patients with stroke [12]. A study in urban New Delhi has shown prevalence of low HDL-C of 37% in the studied population, which was very close to our findings of 44%, as reported by Chandra et al. [13].

In our study, in patients with hypertension, hypocholesterolemia HDL was the most common lipid abnormality unlike Youmbissi et al. who have found that hypercholesterolemia was the most common abnormality followed by hypertriglyceridemia and mixed hyperlipidemia [9]. Tiahou et al. from Ivory Coast found also hypercholesterolemia as the most common lipid abnormality [14] but they carried out a study in all patients who have done a lipid profile at the University Hospital of Cocody during that period and did not consider low HDL cholesterol as an abnormality.

The prevalence of total hypercholesterolemia was 3.4% of patients, which is lower than the one reported by Ndiaye et al. from Senegal (52.34%) in patients with an ischemic stroke; this prevalence is also lower than the one reported by Maiga from Mali in patients with hypertension (23,26%) and the one reported by Goudote from Senegal in patients with coronary artery diseases (19,6%) [15–17]. Hypercholesterolemia is the most common abnormality in patients from Senegal and Ivory Coast [2, 14].

Hypercholesterolemia LDL was found only in 3.8% of our patients; this is very low compared to the percentage found by Apetse et al. in patients with a stroke which was 47,96% in Togo [12] and it is also low compared to the one found by Doupa et al. which was 22,5% in Senegal [2]. We have reported this finding in a previous study, where we have found that hypercholesterolemia was uncommon in patients with ischemic stroke in association with low prevalence of significant carotid stenosis [18]. Rather than hypercholesterolemia in high risk patients, we have found hypocholesterolemia HDL to be very high in our study population and this was the most common abnormality found in 44.3% of the patients. Hypocholesterolemia HDL has been also found to be common in Nigeria in a group of patients with cardiovascular risks as reported by Umar et al. [19]. Gonzalez-Pacheco et al. have also reported a high percentage of patients with hypocholesterolemia HDL (68.6%) in patients with acute coronary syndrome in Mexico [20]. The low level of HDL cholesterol may be due to antilipid medication (only 35 patients were taking anticholesterol medication); we believe that this low HDL cholesterol is a common finding in the lipid profiles of patients in our environment. Low levels of HDL cholesterol are as common as high levels of LDL cholesterol as has been reported in one study in which low level of HDL was the most common abnormality of lipid profile found in the population [21]; although low HDL-C is considered as a dyslipidemia and elevated HDL-C has been found to have cardiovascular protective effect, little is known about the exact clinical relevance of low HDL-C [22].

In general, low levels of HDL cholesterol in human are secondary to type 2 diabetes mellitus and the metabolic syndrome because of the increase catabolism due to hypertriglyceridemia [21]. There are other reported causes of low level of HDL: sedentariness, low fat diet, medications, and drugs; genetic abnormalities are less common [21]. In one study, low HDL cholesterol in association with hypertriglyceridemia has been found in 8% of the patients [23]. The atherogenic index is high when the HDL is low; 36% of our patients had an elevated atherogenic index and 44.3% had a very low HDL level, making us state that low HDL should also be considered as a risk factor for cardiovascular disease in Cameroon as reported [24, 25]. Elevated LDL which is a well-known risk factor is low in our country, while low HDL which is equally a recognized factor is more prevalent.

Hypertriglyceridemia was present in 18.9% of our patients which is more than the prevalence found by Nissaf et al. in Tunisia which was 13.6% [23] and this was lower than the 25% found by Sumner et al. in their patients with a metabolic syndrome [26].

There was a strong association between diabetes and hypertriglyceridemia as has been found by Lokrou in Ivory Coast [27]. There was no association between tobacco and hypertriglyceridemia or alcohol and hypertriglyceridemia as has been reported by Abessolo et al. in Gabon [28].

## 5. Conclusion

In this population with cardiovascular risk factors and diseases, the most common lipid abnormality was low HDL cholesterol. Few patients presented elevated LDL cholesterol compared to the literature, making hypercholesterolemia be considered as a less common cardiovascular risk factor compared to hypertension and low HDL cholesterol which were very common. We would recommend multicenter studies and population based studies to further assess this important finding.

## Figures and Tables

**Table 1:** Lipid profile abnormalities in the studied population.

Parameters	Values (g/L)	Number	Percentage
Total cholesterol	Low ≤ 1.4	42	15.90%
	Normal 1.5–2.7	213	80.70%
	High > 2.7	**9**	**3.40%**

HDL cholesterol	Low ≤ 0.41	**117**	**44.30%**
	Normal 0.41–0.75	131	49.60%
	High > 0.75	16	6.10%

LDL cholesterol	Low ≤ 1	100	37.90%
	Normal 1–1.9	154	58.30%
	High > 1.9	**10**	**3.80%**

Triglycerides	Low < 1	123	46.60%
	Normal 1–1.5	91	34.50%
	High > 1.5	**50**	**18.90%**

Total		264	100.00%

**Table 2:** Comparison of the lipid profile in men and women.

Lipid	Sex	Mean levels	Lowest	Maximum	Standard deviation	*P* value
Total cholesterol	Male	1.78 g/L	0.69 g/L	2.99 g/L	0.43	*P* = 0.01
	Female	1.90 g/L	0.93 g/L	3.37 g/L	0.44	
HDL cholesterol	Male	0.46 g/L	0.14 g/L	1.26 g/L	0.18	*P* = 0.14
	Female	0.49 g/L	0.13 g/L	1.22 g/L	0.19	
LDL cholesterol	Male	1.09 g/L	0.11 g/L	2.4 g/L	0.42	*P* = 0.03
	Female	1.18 g/L	0.2 g/L	2.55 g/L	0.39	
Triglycerides	Male	1.13 g/L	0.40 g/L	3.05 g/L	0.52	*P* = 0.7
	Female	1.15 g/L	0.31 g/L	4.33 g/L	0.55	

**Table 3:** Distribution of dyslipidemia in patients with a low HDL cholesterol.

Low HDL cholesterol				
Triglycerides				
LDL	Low	Normal	High	Total
Low	14 (11.97%)	12 (10.26%)	11 (9.4%)	37 (31.62%)
Normal	28 (23.93%)	26 (22.22%)	22 (18.8%)	76 (64.96%)
High	1 (0.85%)	2 (1.71%)	1 (0.85%)	4 (3.42%)

Total	43	40	34	117

**Table 4:** Distribution of dyslipidemia in patients with a normal level of HDL cholesterol.

Normal HDL cholesterol				
Triglycerides				
LDL	Low	Normal	High	Total
Low	32 (24.43%)	14 (10.69%)	7 (5.34%)	53 (40.46%)
Normal	37 (28.24%)	27 (20.61%)	8 (6.11%)	72 (54.96%)
High	1 (0.76%)	5 (3.82%)	0 (0%)	6 (4.58%)

Total	70	46	15	131

**Table 5:** Distribution of dyslipidemia in patients with a high HDL cholesterol.

High HDL cholesterol				
Triglycerides				
LDL	Low	Normal	High	Total
Low	6 (37.5%)	3 (18.75%)	1 (6.25%)	10
Normal (%)	4 (25%)	2 (12.5%)	0 (0%)	6 (37.5%)

Total	10	5	1	16
